# Primary Small Cell Carcinoma of the Hypopharynx: A Case Report of a Rare Tumor

**DOI:** 10.1155/2015/934926

**Published:** 2015-06-07

**Authors:** Ali Bayram, Ebru Akay, Sema S. Göksu, İbrahim Özcan

**Affiliations:** ^1^Department of ENT, Kayseri Training and Research Hospital, Sanayi Mahallesi, Atatürk Bulvari Hastane Caddesi No. 78, Kocasinan, 38010 Kayseri, Turkey; ^2^Department of Pathology, Kayseri Training and Research Hospital, Sanayi Mahallesi, Atatürk Bulvari Hastane Caddesi No. 78, Kocasinan, 38010 Kayseri, Turkey; ^3^Department of Medical Oncology, Kayseri Training and Research Hospital, Sanayi Mahallesi, Atatürk Bulvari Hastane Caddesi No. 78, Kocasinan, 38010 Kayseri, Turkey

## Abstract

*Introduction*. Primary hypopharynx involvement of small cell carcinoma is very rare and very few cases have been reported in the literature. Here, we report a case of primary small cell carcinoma of the hypopharynx in a male patient. *Case Report*. A 50-year-old man presented with a 6-month history of sore throat and swellings in the right side of the neck. Direct laryngoscopy and biopsy revealed small cell carcinoma of the hypopharynx located in the right pyriform sinus. *Discussion*. Small cell carcinoma of the hypopharynx has no clear treatment modality due to the rarity of the disease. Systemic chemotherapy and radiotherapy should have priority among the therapy regimens because of the high metastatic potential of the tumor.

## 1. Introduction

Small cell carcinoma (SMCC) is a member of the neuroendocrine tumors, which most frequently occurs in the lungs. Extrapulmonary SMCC constitutes 2.5% to 5% of all SMCCs [1]. These carcinomas are rarely seen in the head-neck region with laryngeal predominance, and the prognosis is very poor [2]. Primary hypopharynx involvement is extremely rare and very few cases have been reported [3–5]. Here, we report a case of primary SMCC of the hypopharynx in a male patient.

## 2. Case Report

**Figure 1:**
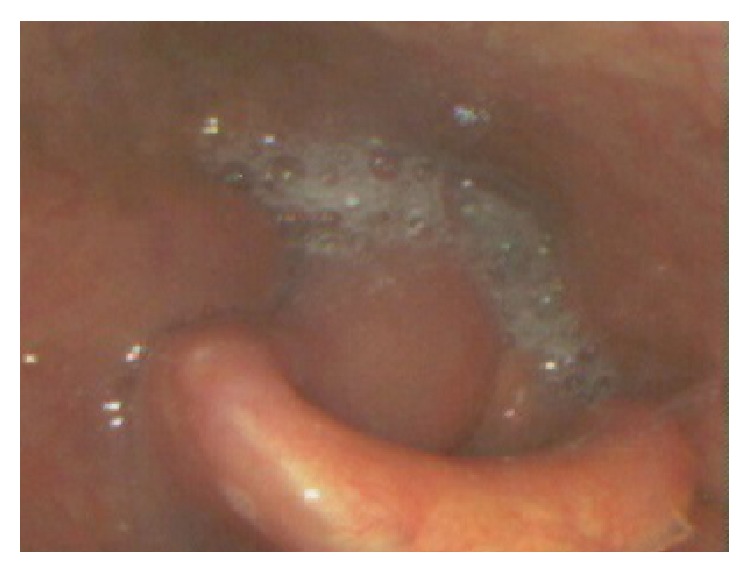
Bilateral intense arytenoid edema with fullness of the right pyriform sinus.

A 50-year-old male patient presented with a history of sore throat and neck swellings persisting for about six months. There was no history of dyspnea or weight loss. He had a 60-pack-year history of smoking with no alcohol consumption. Flexible laryngoscopy under local anesthesia revealed bilateral intense arytenoid edema with fullness of the right pyriform sinus area. There were multiple right-sided palpable lymph nodes. The patient was scheduled for direct laryngoscopy and biopsy under general anesthesia. On the day of admission, the patient left the hospital in spite of detailed information given about the disease and the risks. He refused further interventions, including radiological investigations. One month later, he presented to the emergency unit with severe respiratory distress and was immediately taken to the operating room. The airway was secured via tracheotomy and direct laryngoscopy revealed an ulcerovegetant tumoral lesion originating from the medial wall of the right pyriform sinus. The tumor was filling the entire sinus and multiple punch biopsies were taken from the tumor. The remaining laryngeal structures had an intense edematous appearance (Figure 1).

**Figure 2:**
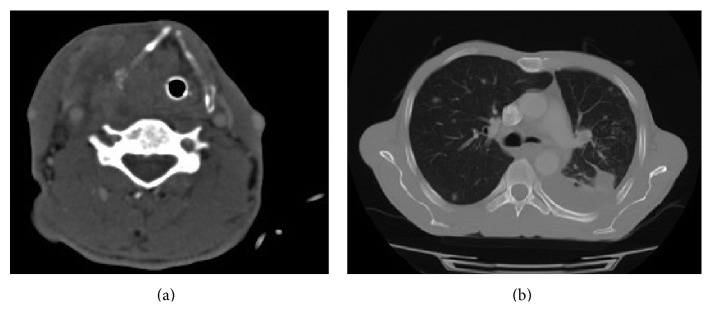
(a) CT scan with contrast enhancement showing a tumoral mass of the right pyriform sinus extending through the extralaryngeal region with thyroid cartilage destruction and prevertebral area involvement. (b) CT scan demonstrating multiple metastases in both lungs.

Contrast-enhanced CT (Figure 2(a)) and MRI examinations demonstrated a tumoral mass at the right pyriform sinus extending through the extralaryngeal region with thyroid cartilage destruction and prevertebral area involvement. There were multiple lymphadenopathies with pathological configuration in the right side of the neck. Multiple metastatic nodules were detected in both lungs with thorax CT (Figure 2(b)). Radiological studies showed no other distant metastatic disease.

**Figure 3:**
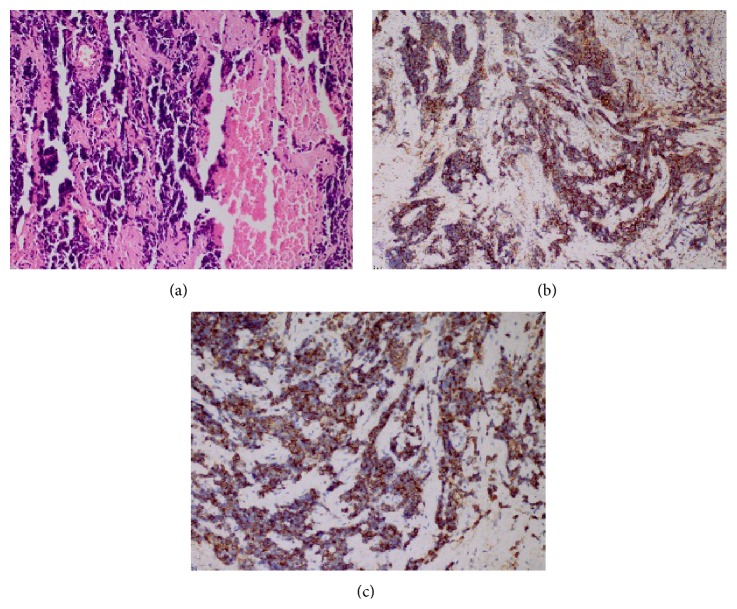
(a) Hematoxylin and eosin staining showing small cells, round to fusiform in shape with scanty cytoplasm, fine granular nuclear chromatin, and absence of nucleoli. Note the necrosis and crush artifact (original magnification ×200). The tumor cells were immunoreactive for (b) pancytokeratin and (c) CD56 (original magnification ×200).

Histopathological analysis of the biopsy revealed a tumor consisting of tightly packed nests and diffuse irregularly shaped sheets of cells with areas of necrosis. The tumor cells were of small size with hyperchromatic, round to oval nuclei and scanty, poorly defined cytoplasm (Figure 3(a)). The nuclear chromatin was finely granular and nucleoli were absent. Nuclear molding was prominent and a typical crush artifact was present. The tumor cells were immunoreactive for pancytokeratin (Figure 3(b)), CD56 (Figure 3(c)), focal synaptophysin, and thyroid transcription factor- (TTF-) 1 but negative for CD20, leukocyte common antigen, and S100. The Ki-67 index was high (more than 70%). These findings were compatible with primary oat cell SMCC of the hypopharynx.

Three courses of chemotherapy, composed of cisplatin (CDDP) (75 mg/m^2^, day 1) and etoposide (100 mg/m^2^, days 1–3) (VP-16) on days 1–3, were administered to the patient for the initial treatment, which is similar to the standard regimen for lung SMCC in our hospital. After completion of chemotherapy, complete response was achieved at the primary tumor site and the neck. Metastatic nodules in the lungs also disappeared and the treatment was maintained with radiotherapy to the primary tumor and right side of the neck. Radiotherapy was performed by simultaneous integrated boost fractionation at 2.12 Gy/fraction/day. After the 16th session, the patient refused further treatment. Complete physical and radiological examination revealed no evidence of residual tumor although there was early cessation of radiotherapy. Decannulation was achieved during the follow-up and the patient has remained free of disease for 15 months.

## 3. Discussion 

SMCC of the hypopharynx is an extremely rare condition, but a definitive diagnosis is essential to determine the appropriate treatment plan. SMCC has three different histopathological types, including oat cell, intermediate-cell, and combined cell. It is characterized by small cells with hyperchromatic nuclei and scanty cytoplasm. Cell necrosis and mitotic activity are frequent within the tumor [6]. Immunohistochemical studies are necessary to confirm the diagnosis. SMCC may express positivity for cytokeratins, epithelial membrane antigen, carcinoembryonic antigen, and neuroendocrine markers, including neuron-specific enolase, CD56, CD57, chromogranin, synaptophysin, neuropeptides, including calcitonin, somatostatin, adrenocorticotropic hormone, bombesin, and serotonin. SMCC may also be immunoreactive for TTF-1 [7]. The pathological type of the present case was oat cell carcinoma and the tumor cells were immunoreactive for pancytokeratin and CD56 and focally immunoreactive for synaptophysin and TTF-1.

SMCC is a very aggressive neoplasm, which metastasizes to cervical lymph nodes, bone, skin, liver, and lung in the early period of the disease. Cervical lymph node metastases occur in about half of the patients and more than 90% of patients develop metastatic disease [8]. The aggressive nature of this tumor reduces the 5-year survival rate to as low as 5%. Treatment of SMCC of the head and neck region is based on that of pulmonary SMCC, due to the rarity of head-neck involvement. Surgical procedures have failed in the majority of cases that have been reported in the larynx [9]. In a reported case of combined SMCC of the hypopharynx that was treated with surgery alone, the patient died nine months after the initial therapy due to distant metastasis in spite of an additional chemotherapy regimen [3]. Gaba et al. reported a case of hypopharyngeal SMCC that was successfully treated with concurrent platinum-based chemotherapy and neck irradiation [4]. Sano et al. achieved complete response with a combination treatment composed of systemic chemotherapy and radiotherapy in a case of primary hypopharyngeal SMCC; however, the patient died due to lung and liver metastases that occurred one month after the treatment [5]. Multimodality treatment of SMCC, including radiotherapy combined with chemotherapy, seems to be a rational therapeutic option because of the potential of distant metastasis of the disease. In the present case, the patient was initially treated with chemotherapy because of the presence of multiple metastases in the lungs. After three courses of chemotherapy composed of CDDP and VP-16, metastatic pulmonary nodules disappeared and complete response was achieved at the primary tumor site. The treatment was maintained with radiotherapy to the primary tumor and right side of the neck by simultaneous integrated boost fractionation at 2.12 Gy/fraction/day but the patient refused further treatment after the 16th session. Although there was early cessation of radiotherapy, the patient had complete remission. Close follow-up is still ongoing and the patient has remained free of tumor for 15 months.

## 4. Conclusion

SMCC of the hypopharynx is an extremely rare tumor in the head-neck region with no clear treatment modality. Multimodality treatment with systemic chemotherapy and radiotherapy should have priority among therapy regimens due to the high metastatic potential of the tumor.

## References

[1] Renner G. (2007). Small cell carcinoma of the head and neck: a review. *Seminars in Oncology*.

[2] Ferlito A., Devaney K. O., Rinaldo A. (2006). Neuroendocrine neoplasms of the larynx: advances in identification, understanding, and management. *Oral Oncology*.

[3] Uwa N., Terada T., Mohri T. (2013). Combined small cell carcinoma of the hypopharynx. *Auris Nasus Larynx*.

[4] Gaba A., Mbaoma R., Breining D., Smith R. V., Beitler J. J., Haigentz M. (2005). Unusual sites of malignancies: case 1. Small-cell carcinoma of the hypopharynx. *Journal of Clinical Oncology*.

[5] Sano M., Kitahara N., Toma M. (2005). Hypopharyngeal small cell carcinoma: a case report. *Auris Nasus Larynx*.

[6] Aggarwal G., Jackson L., Sharma S. (2011). Primary combined small cell carcinoma of larynx with lateralized histologic components and corresponding sides-pecific neck nodal metastasis: report of a unique case and review of literature. *International Journal of Clinical and Experimental Pathology*.

[7] Mikić A., Zvrko E., Trivić A., Stefanović D., Golubović M. (2013). Small cell neuroendocrine tumor of the larynx—a small case series. *Collegium Antropologicum*.

[8] Gnepp D. R. (1991). Small cell neuroendocrine carcinoma of the larynx. A critical review of the literature. *ORL*.

[9] Ferlito A., Rinaldo A. (2008). Primary and secondary small cell neuroendocrine carcinoma of the larynx: a review. *Head and Neck*.

